# Vaccination against Endogenous Retrotransposable Element Consensus Sequences Does Not Protect Rhesus Macaques from SIVsmE660 Infection and Replication

**DOI:** 10.1371/journal.pone.0092012

**Published:** 2014-03-20

**Authors:** Neil C. Sheppard, R. Brad Jones, Benjamin J. Burwitz, Francesca A. Nimityongskul, Laura P. Newman, Matthew B. Buechler, Jason S. Reed, Shari M. Piaskowski, Kim L. Weisgrau, Philip A. Castrovinci, Nancy A. Wilson, Mario A. Ostrowski, Byung Park, Douglas F. Nixon, Eva G. Rakasz, Jonah B. Sacha

**Affiliations:** 1 Vaccine Research, Worldwide R&D, Pfizer Inc., San Diego, California, United States of America; 2 Department of Immunology, University of Toronto, Toronto, ON, Canada; 3 Vaccine and Gene Therapy Institute, Oregon Health & Science University, Beaverton, Oregon, United States of America; 4 Oregon National Primate Research Center, Oregon Health & Science University, Beaverton, Oregon, United States of America; 5 Wisconsin National Primate Center, University of Wisconsin, Madison, Wisconsin, United States of America; 6 Division of Experiment Medicine, San Francisco General Hospital, University of California San Francisco, San Francisco, California, United States of America; 7 Department of Medical Microbiology and Immunology, Oregon Health & Science University, Portland, Oregon, United States of America; 8 Division of Biostatistics, Department of Public Health and Preventive Medicine, Oregon Health and Science University, Portland, Oregon, United States of America; Tulane University, United States of America

## Abstract

The enormous sequence diversity of HIV remains a major roadblock to the development of a prophylactic vaccine and new approaches to induce protective immunity are needed. Endogenous retrotransposable elements (ERE) such as endogenous retrovirus K (ERV)-K and long interspersed nuclear element-1 (LINE-1) are activated during HIV-1-infection and could represent stable, surrogate targets to eliminate HIV-1-infected cells. Here, we explored the hypothesis that vaccination against ERE would protect macaques from acquisition and replication of simian immunodeficiency virus (SIV). Following vaccination with antigens derived from LINE-1 and ERV-K consensus sequences, animals mounted immune responses that failed to delay acquisition of SIVsmE660. We observed no differences in acute or set point viral loads between ERE-vaccinated and control animals suggesting that ERE-specific responses were not protective. Indeed, ERE-specific T cells failed to expand anamnestically *in vivo* following infection with SIVsmE660 and did not recognize SIV-infected targets *in vitro*, in agreement with no significant induction of targeted ERE mRNA by SIV in macaque CD4+ T cells. Instead, lower infection rates and viral loads correlated significantly to protective *TRIM5*α alleles. Cumulatively, these data demonstrate that vaccination against the selected ERE consensus sequences in macaques did not lead to immune-mediated recognition and killing of SIV-infected cells, as has been shown for HIV-infected human cells using patient-derived HERV-K-specific T cells. Thus, further research is required to identify the specific nonhuman primate EREs and retroviruses that recapitulate the activity of HIV-1 in human cells. These results also highlight the complexity in translating observations of the interplay between HIV-1 and human EREs to animal models.

## Introduction

HIV vaccine research has yielded numerous novel strategies. However, these approaches have yet to effectively address the enormous amount of HIV sequence diversity present within and between infected hosts. Invariable surrogate markers of HIV infection would provide a “static” vaccine target, and immune responses engendered against such targets could theoretically provide lasting control of HIV replication. There is growing evidence and appreciation of the interaction between germline endogenous retrotransposable elements (ERE) and exogenous retroviruses in multiple species. In mice, infection with exogenous ectopic MuLV results in replication of defective endogenous polytopic retroviruses [1]. We, and others, have observed that certain ERE including specific human endogenous retrovirus (HERV) families [2]–[11] and long interspersed nuclear element (LINE)-1 insertions [12], which normally remain dormant in healthy somatic cells, are activated following HIV infection. HIV-induced activation of these EREs not only triggers detectable immune responses that inversely correlate with control of HIV viremia [5]–[7], but HERV-K-specific T cell clones isolated from HIV-infected patients directly recognize and kill HIV-infected cells *in vitro* [8]. Inducing similar immune responses directed to HERV-K, and also LINE-1, by vaccination thus represents a novel HIV vaccine strategy, but one that faces complex hurdles regarding safety and immunogenicity due to the potential for recognition of self-antigens.

Previously, we conducted studies in mice and Indian rhesus macaques to address the hurdles of safety and immunogenicity [13]. Having safely induced polyfunctional T cell responses in macaques to antigens representing consensus sequences of Simian ERV (SERV)-K Gag and Env, and human LINE1, in addition to antibody responses to SERV-K Env, we proceeded here to examine whether the rhesus SIV-infected macaque model of HIV might be suitable to assess the efficacy of immune responses against the selected EREs in preventing or controlling SIV infection. In support of the potential suitability of the model, we previously found that SIV activates HERV-K in human CD4+ T cells, allowing recognition by HERV-K Env- and Pol-specific CD8+ T cell clones [8]. Thus, SIV has the ability to activate these specific EREs in the human genome. Moreover, it is known that ERV-K [14],[15] and LINE-1 [16]–[18] share common ancestors prior to speciation of modern primates and humans.

To address the efficacy of our ERE-based vaccine, we challenged vaccinated animals intra-rectally with low-dose SIVsmE660 and monitored subsequent viremia. No vaccine-induced protection against SIVsmE660 acquisition was observed. Moreover, ERE-specific T cells failed to expand anamnestically in response to SIV infection, indicating that SIV did not induce expression of SERV-K or LINE-1 antigens corresponding to our vaccine inserts in infected rhesus macaque cells. Consequently, we observed no differences in acute or chronic phase viral loads between the vaccine and sham-vaccinated groups. Compared to HIV infection of human cells [8] we found only marginal induction of SERV-K transcripts at the mRNA level by SIV in *ex vivo* infections. Since control of ERE expression can occur post-transcriptionally [19]–[25], we also tested the ability of SERV-K- and LINE-1-specific T cells to recognize SIV-infected cells *in vitro*. In agreement with our previous results, we found that ERE-specific T cells did not respond to SIV-infected target cells. Therefore, while vaccination against ERE appears safe and immunogenic in mice and nonhuman primates, SIVsmE660-infected Indian rhesus macaques are not an appropriate model for establishing the efficacy of immunization with SERV-K Gag and Env and LINE-1 since SIVsmE660 does not induce expression of these elements in the rhesus macaque genome. Alternative models that more closely mirror the induction of ERE in humans by HIV must be established in order to address efficacy before ERE-based vaccines can move forward for use as a prophylactic or therapeutic HIV vaccine approach.

## Materials and Methods

### Animals

Twenty-four Indian rhesus macaques (*Macaca mulatta*) housed at the Wisconsin National Primate Research Center (WNPRC) were used in this study and immunized as described previously [13]. The University of Wisconsin Institutional Animal Care and Use Committee (animal welfare assurance no. A3368-01) reviewed and approved all study protocols. All macaques in this study were managed according to the WNPRC animal husbandry program, which aims at providing consistent and excellent care to nonhuman primates. This program is based on the laws, regulations, and guidelines set forth by the United States Department of Agriculture (e.g., the Animal Welfare Act and its regulations, and the Animal Care Policy Manual), Institute for Laboratory Animal Research (e.g., Guide for the Care and Use of Laboratory Animals, 8^th^ edition), Public Health Service, National Research Council, Centers for Disease Control, and the Association for Assessment and Accreditation of Laboratory Animal Care (AAALAC) International. The nutritional plan utilized by the WNPRC is based on National Research Council recommendations. Specifically, macaques were fed twice daily with 2050 Teklad Global 20% Protein Primate Diet and food intake was closely monitored by Animal Research Technicians. This diet was supplemented with a variety of fruits, vegetables, and other edible objects as part of the environmental enrichment program established by the Behavioral Management Unit. Paired/grouped animals exhibiting incompatible behaviors were reported to the Behavioral Management staff and managed accordingly. All primary enclosures and animal rooms were cleaned daily with water and sanitized at least once every two weeks. All efforts were made to minimize suffering through the use of minimally invasive procedures, anesthetics, and analgesics when appropriate. Animals were painlessly euthanized with sodium pentobarbital and euthanasia was assured by exsanguination and bilateral pneumothorax, consistent with the recommendations of the American Veterinary Medical Guidelines on Euthanasia (June, 2007). Animals used in this study were typed for the MHC-I alleles Mamu-A*01, *Mamu*-A*02, *Mamu*-A*08, *Mamu*-A*11, *Mamu*-B*01, *Mamu*-B*03, *Mamu*-B*04, *Mamu*-B*08, *Mamu*-B*17, and *Mamu*-B*29 using sequence-specific priming PCR (PCR-SSP) as previously described [26], [27]. We excluded Mamu-B*17 ^+^ and Mamu-B*08 ^+^ animals from this study because these alleles are associated with spontaneous control of SIV replication. The animals were also genotyped for the following three allelic *TRIM5* classes: *TRIM5^CypA^, TRIM5^TFP^, and TRIM5^Q^* as previously described [28].

### Expression of SERV-K and L1 mRNA in SIV-Infected CD4 T Cells

Peripheral blood mononuclear cells (PBMC) were isolated from whole blood samples of SIV-uninfected rhesus macaques by Ficoll gradient centrifugation: r94048, r99037, r99002, r99055 and r96022 were undergoing a safety and immunogenicity study as previously described [13]. CD4+ T cells were isolated using anti-macaque CD4 beads (Miltenyi, Auburn, CA]) according to manufacturer’s instructions. The CD4 enriched population was activated by addition of SEB, anti-CD3, anti-CD28 and anti-CD49d to the R15–100 medium (RPMI, 15% FBS, 100 U/mL human IL-2) for 24 h before replacement of the medium with R15–100 alone, as previously described [29]. The cells were cultured for a further 6 days, with medium changed as necessary. 75% of the CD4 enriched cells were then infected with SIVsmE660 by magnetofection (OZ Biosciences), while the remaining 25% were left uninfected as controls. Time points were taken at 0 h, 72 h and 96 h. 2×10^5^ cells were taken at each time point post infection to measure SIV Gag p27 expression by flow cytometry. At 72 h and 96 h post infection, 3×10^7^ cells of each type were harvested. The SIV-infected CD4+ T cells were CD4-depleted by MACS to enrich for SIV-infected cells (which down-regulate CD4 surface expression), and the CD4-depleted (SIV-enriched) fraction lysed in RLT buffer (Qiagen, Valencia, CA) plus 2-ME. The CD4-enriched fraction was returned to culture. Lysed samples (up to 10 million cells per 600 μL of RLT) were frozen at −80°C. Uninfected cells were also lysed at each time point as controls. Total RNA was isolated using the All-Prep kit (Qiagen, Valencia, CA), following genomic DNA (gDNA) isolations, using manfacturer’s instructions. mRNA was amplified from the samples using the MessageAmp II aRNA amplification kit (Ambion, Austin TX). Amplified mRNA was then treated with Turbo DNAse (Ambion, Austin TX). Quantitative PCR reactions were performed with 100 ng of amplified RNA per 10ul reaction in 384 well plates using the Power SYBR Green RNA-to-C_T_ kit (Applied Biosystems, Foster City CA) and the following cycling conditions: 48°C –30 min, 95°C –10 min, 40 cycles of (95°C –15 sec, 58°C –1 min) on an ABI Prism 7900HT Sequence Detection System (Applied Biosystems) Products from initial reactions were sequenced to confirm identity, and melting curve analyses were performed to assess the uniformity of these products. For all the SIV, ERV-K, and SRVmac amplicons, absolute quantities of products were determined by referencing C_T_ values to standard curves generated using plasmids containing target sequences (see Table S2) that had been serially diluted at known copy numbers. For L1 amplicons, relative quantities were determined using a standard curve of serially diluted rhesus gDNA. Primer sequences are given in Table S3. The quantities of each amplicon are expressed as a ratio to TBP mRNA.

### Viral Infection

All animals were challenged intrarectally (IR) with up to 5 inoculations of 6×10^6^ viral RNA copies of SIVsmE660 (225 50% tissue culture infectious doses (TCID_50_)) on a weekly basis until infected. If an animal remained uninfected after these 5 inoculations, then up to an additional 2 IR inoculations of 1.2×10^7^ copies of SIVsmE660 (450 TCID_50_) were administered on a weekly basis until the animal became infected. If animals still remained uninfected, then one additional IR inoculation of 1.2×10^8^ copies of SIVsmE660 (4,500 TCID_50_) was administered. If animals remained uninfected following this final IR dose, animals were then challenged intravenously (IV) with 100 ng of SIVsmE660 Gag p27^CA^. One control animal, r07045, remained uninfected following all IR and IV challenge. Animals were considered positive for SIV infection after at least two subsequent positive viral load determinations and were then no longer challenged.

### Viral Load Determination

Levels of circulating plasma virus were determined using a previously described quantitative reverse transcription-PCR assay that detects SIVsmE660 [30]. Virus concentrations were determined by interpolation onto a standard curve of *in vitro*-transcribed RNA standards in serial 10-fold dilutions using a LightCycler (Roche).

#### IFN-γ ELISPOT and peptides

Fresh peripheral blood mononuclear cells (PBMC) isolated from EDTA-anticoagulated blood were used for the detection of IFN-γ secreting cells as previously described with the exception of how positive responses were determined [31]. Test wells were run with two replicates, while control wells were run with replicates of 2, 4, or 6, depending on the assay. Positive responses were determined using a one-tailed *t* test and an alpha level of 0.05, where the null hypothesis was that the background level would be greater than or equal to the treatment level. If determined to be positive statistically, the values were reported as the average of the test wells minus the average of all negative-control wells. Two peptide sets were obtained from Pepscan (Lelystad, Netherlands): 15-mers overlapping by 11 amino acids spanning the entire SERV-K Gag and Env proteins and peptides predicted to bind the Mamu-A*01 and –A*02 MHC-I molecules. For the predicted peptides, we performed *in silico* studies of epitope prediction using the MHCPathway Macaque algorithm (www.mamu.liai.org) and the amino acid sequence for human LINE-1 Open Reading Frame 2 (ORF 2) and SERV-K Gag and Env. For LINE-1 ORF 2, 15-mer peptides overlapping by 11 amino acids were obtained from JPT (Berlin, Germany).

### T Cell Lines and in vitro Recognition Assays

CD8+ and CD4+ T cell lines were generated as previously described [29] [32]. Briefly, freshly isolated PBMC from animals making the response of interest were co-cultured at a ratio of 1∶1 with autologous, lethally-irradiated B lymphoblastoid cells (BLCL) pulsed with the peptide of interest in R15–100 media (RPMI 1640 medium containing 15% FCS and 100 U/ml IL-2). IL-2 was obtained through the National Institute of Health (NIH) AIDS Reagent Program. Cell lines were maintained in R15–100 media and stimulated weekly with peptide-pulsed BLCL as described above. Autologous CD4+ T cell and macrophage targets were generated and infected with SIVmac239, SIVmac316E, or SIVsmE660 as described previously [29], [32], [33]. Twenty-four hours following infection, productive SIV infection was confirmed performing intracellular Gag p27 staining as described previously [29]. Productively infected target cells were then used as antigen presenting cells and co-cultured with ERE-specific T cells for 6.5 hours. Following this incubation, intracellular cytokine staining (ICS) was performed and positive recognition determined as previously described [29].

### Statistics

To measure for differences in viral loads between vaccine and control groups, we used repeated measures of ANOVA. Since in a typical experiment using repeated measures, two measurements taken at adjacent times are more highly correlated than two measurements taken several time points apart, we used a Bayesian Information Criterion (BIC) to determine the optimal correlation. Pre-planned contrasts were used to compare the difference in viral loads between control and vaccinated groups at each weeks post infection (WPI). Prior to applying the repeated measures ANOVA, the viral load data was transformed using a logarithm function with base 10 to hold normal distribution assumption. P-values were adjusted by the Benjamini & Hochberg False Discovery Rate (FDR) procedure, which was also applied to comparisons of ERE mRNA levels with and without SIV infection. Statistical significance was determined at the level of 0.05. Area under the curve (AUC) as an overall measure of viremia was calculated by Trapezoidal Integration. One-way ANOVA was used for assessing difference in AUC between control and vaccinated groups. Prior to one-way ANOVA, Levene’s test for homogeneous variance was performed. To analyze the number of challenges needed for infection, we used a generalized gamma model. The best model among gamma, poisson, multinomial, and normal was determined by using Akaike information criterion. We used the Kaplan-Meier method to test whether any of the groups differed in the number of challenges required to achieve productive infection. All analyses were performed using SAS 9.3.

## Results

### ERE Vaccination does not Protect against SIVsmE660 Acquisition or Replication

**Figure 1:**
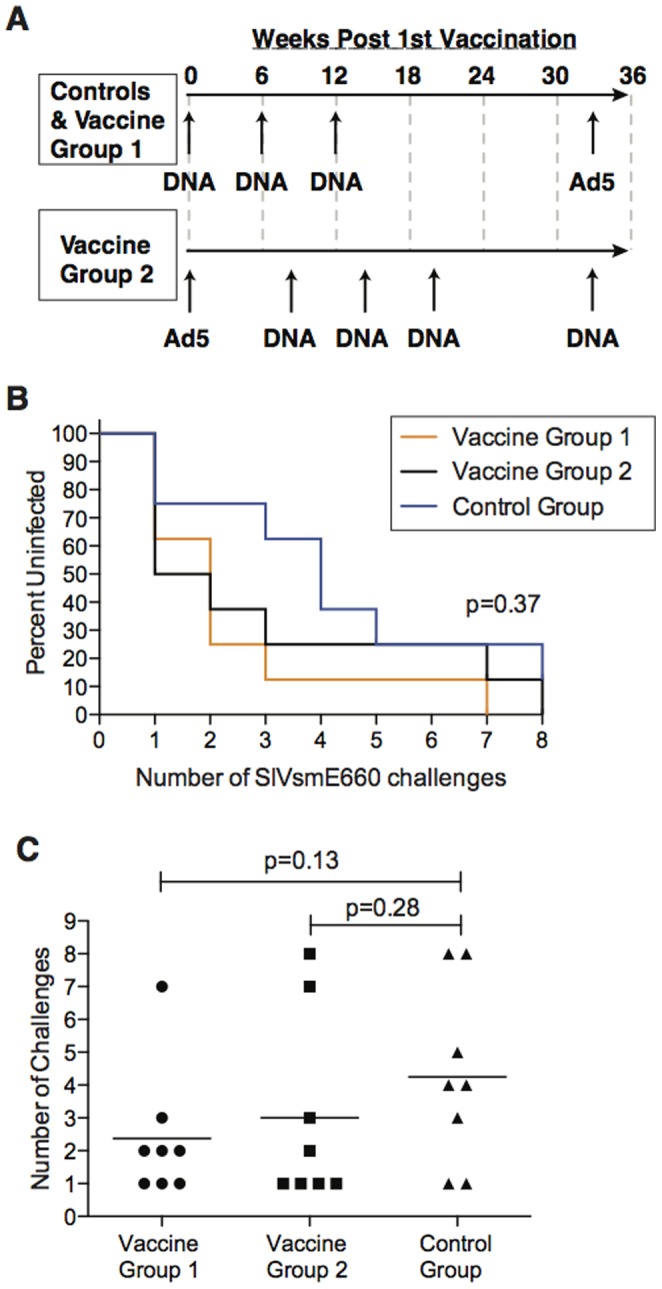
ERE vaccination does not protect Indian rhesus macaques from SIVsmE660 acquisition. [**A**] Vaccine timeline for study. Controls and vaccine group 1 received three DNA primes followed by an rAd5 boost. Vaccine group 2 received an rAd5 prime followed by four DNA boosts. Low dose SIV challenge began at week 36 post first vaccination. [**B**] Kaplan-Meyer curve analysis of the effect ERE vaccination had on the rate of acquisition of SIVsmE660 infection after repeated limiting-dose intrarectal challenge. The statistical significance of the rate of infection was determined by log rank test. No statistically significant effect was observed. [**C**] Comparison of the number of challenges needed to productively infect animals in the vaccine groups and control group with SIVsmE660. The statistical significance of the number of challenges required between the groups was performed by generalized gamma model. Note that one control animal, r07045, remained uninfected throughout the study following seven i.r. and one i.v. challenge, with SIVsmE660. Thus for the purposes of this analysis, the animal was considered as infected after eight challenges. No statistically significant difference was noted.

We recently described the immunogenicity and safety of a vaccine engendering responses against antigens representing consensus sequences of human LINE-1 open reading frame 2 (ORF2) and SERV-K Gag and Env in Indian rhesus macaques [13]. In this study, we randomly assigned twenty-four Indian rhesus macaques into three groups of eight animals each, excluding animals expressing the Major Histocompatibility Complex Class I (MHC-I) molecules Mamu-B*17 and Mamu-B*08 as they are strongly associated with spontaneous control of SIV [27] [34]. The control group received an empty plasmid particle mediated epidermal delivery (PMED) DNA prime followed by a rAd5 boost expressing eGFP (Table S1). Vaccine group one received a SERV-K Gag, SERV-K Env, and human LINE-1 ORF2 PMED DNA prime/rAd5 boost regimen, and vaccine group two received a reversed modality rAd5 prime/multiple PMED DNA boost regimen encoding the same antigens as vaccine group one (Fig. 1A). Vaccination generated humoral immunity against SERV-K Env and cellular immune responses against SERV-K -Gag and -Env and LINE-1 in all animals in vaccine groups one and two, as previously described [13].

To determine if a vaccine targeting ERE such as LINE1 ORF2 and SERV-K might protect against infection with an immunodeficiency virus, we elected to challenge the animals with a low dose, intra-rectal (IR) SIVsmE660 challenge. SIVsmE660 is a swarm virus and challenging animals mucosally with a limiting dose of virus results in transmission of only one to two viral variants, thus more accurately mirroring how humans are infected with HIV compared to high dose challenges [35] [36]. Therefore, we challenged animals weekly and monitored for infection by the presence of plasma viremia. We observed no statistically significant difference in the time to infection (Fig. 1B) or the number of challenges required to infect the animals (Fig. 1C) regardless of vaccine status. Of note, there was a trend towards delayed acquisition in the control group that did not reach statistical significance, which was due to the presence of protective *TRIM5* alleles (see below). Further, one control animal, r07045, never became infected following five low dose IR challenges, one medium dose IR challenge, one high dose IR challenge, and IV challenge with SIVsmE660 (Table S1). Overall, it appeared that the ERE vaccine afforded no protection against acquisition of SIVsmE660.

**Figure 2:**
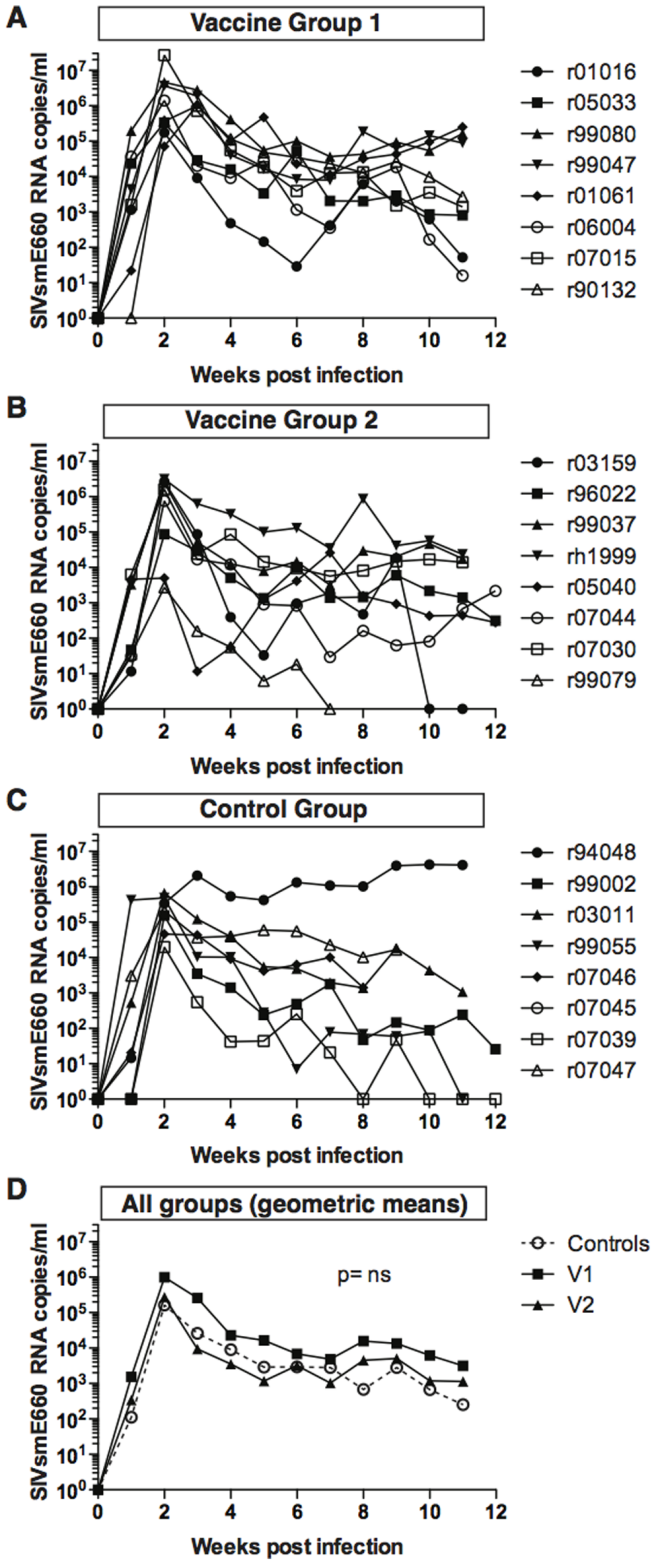
ERE vaccination does not protect Indian rhesus macaques from SIVsmE660 replication. SIVsmE660 plasma viral loads are shown for each animal in [**A**] vaccine group one, [**B**] vaccine group two, and [**C**] the control group. Note that no viral loads exist for animal r07045. [**D**] The geometric mean of the viral loads of each group is shown. No statistically different value was observed between the groups at any time point, as measured by the area under of the curve. The statistical difference between the groups in area under of curve was performed by one-way ANOVA.

Next, we investigated whether the vaccine induced SERV-K –Gag and -Env, and LINE-1-specific T cells blunted peak or set point viral replication. To this end, we followed the plasma viral load of each animal in vaccine group one (Fig. 2A), vaccine group two (Fig. 2B), and the control group (Fig. 2C) throughout the first 12 weeks following infection. There was extreme variability in the viral loads of all animals, due in part to the presence of protective *TRIM5* alleles (see below). Nevertheless, we found no significant difference at any time point in the geometric means of the viral loads between the vaccinated and control groups during acute or set-point viral loads following SIVsmE660 infection (Fig. 2D). Thus, the ERE vaccine did not reduce viral loads following infection.

### Vaccine-elicited T Cells Specific for SERV-K Gag, SERV-K Env, or LINE-1 do not Recognize SIVsmE660 Infected Cells

**Figure 3:**
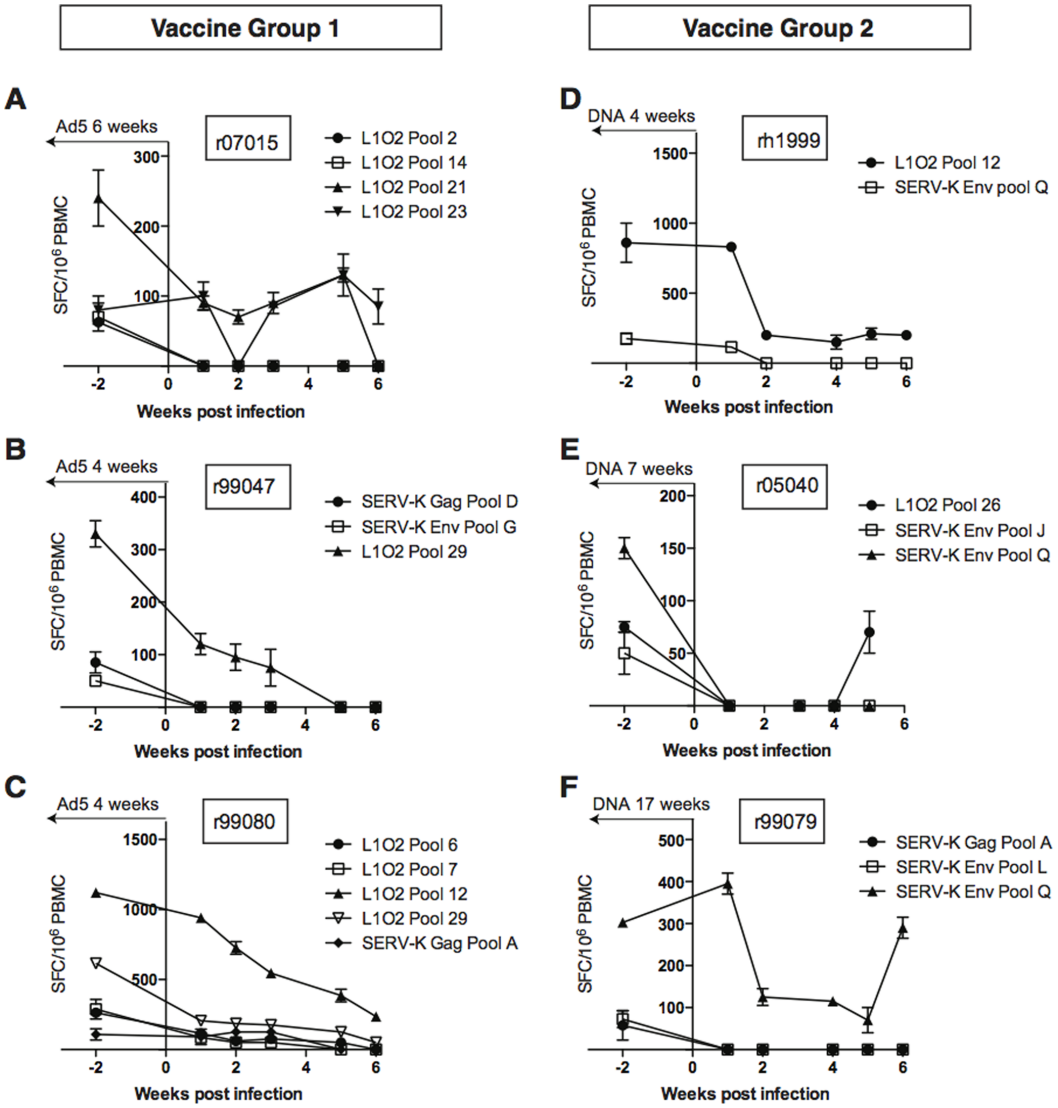
ERE vaccine-induced T cells do not expand *in vivo* following SIVsmE660 infection. Vaccine induced T cell responses detected in ELISPOT above the threshold of 50 IFN-γ spot forming cells (SFCs) at two weeks prior to SIV infection were tracked for the first six weeks post infection and are shown for animals [**A**] r07015, [**B**] r99047, and [**C**] r99080 from vaccine group one and [**D**] rh1999, [**E**] r05040, and [**F**] r99079 from vaccine group two. Similar results were obtained for the remaining animals in both groups. The results shown indicate the mean plus standard deviation of duplicate wells for the indicated peptide pools with the background level subtracted. Time from last ERE vaccination [Ad5 for group 1, DNA for group 2] is indicated at the top of each graph.

Given the lack of protection against SIVsmE660 acquisition and replication in vaccinated animals, we hypothesized that SIVsmE660 infection in rhesus macaques did not recapitulate the ability of HIV to activate ERE expression in human cells. Thus, we anticipated that cellular immune responses in ERE-vaccinated animals would be unable to respond to SIVsmE660-infected targets. To investigate this, we compared the frequency of SERV-K Gag-, SERV-K Env-, and LINE-1-specific cellular immune responses pre- and post-infection by IFN-γ ELISpot. We longitudinally followed responses that were detected above our limit of detection (50 spot forming cells/1 million PBMC) two weeks prior to SIVsmE660 infection. Animal r96022 did not have any detectable responses above our limit of detection at this time point and two animals (r01016 and r99037) had high backgrounds of IFN-γ secretion and therefore were removed from our analysis. We found little to no expansion of vaccine-induced responses following SIVsmE660 infection in either vaccine group (Figure 3 and data not shown). Thus, it appeared that the T cell response engendered by the ERE vaccine did not specifically respond to SIVsmE660-infected cells *in vivo*.

**Figure 4:**
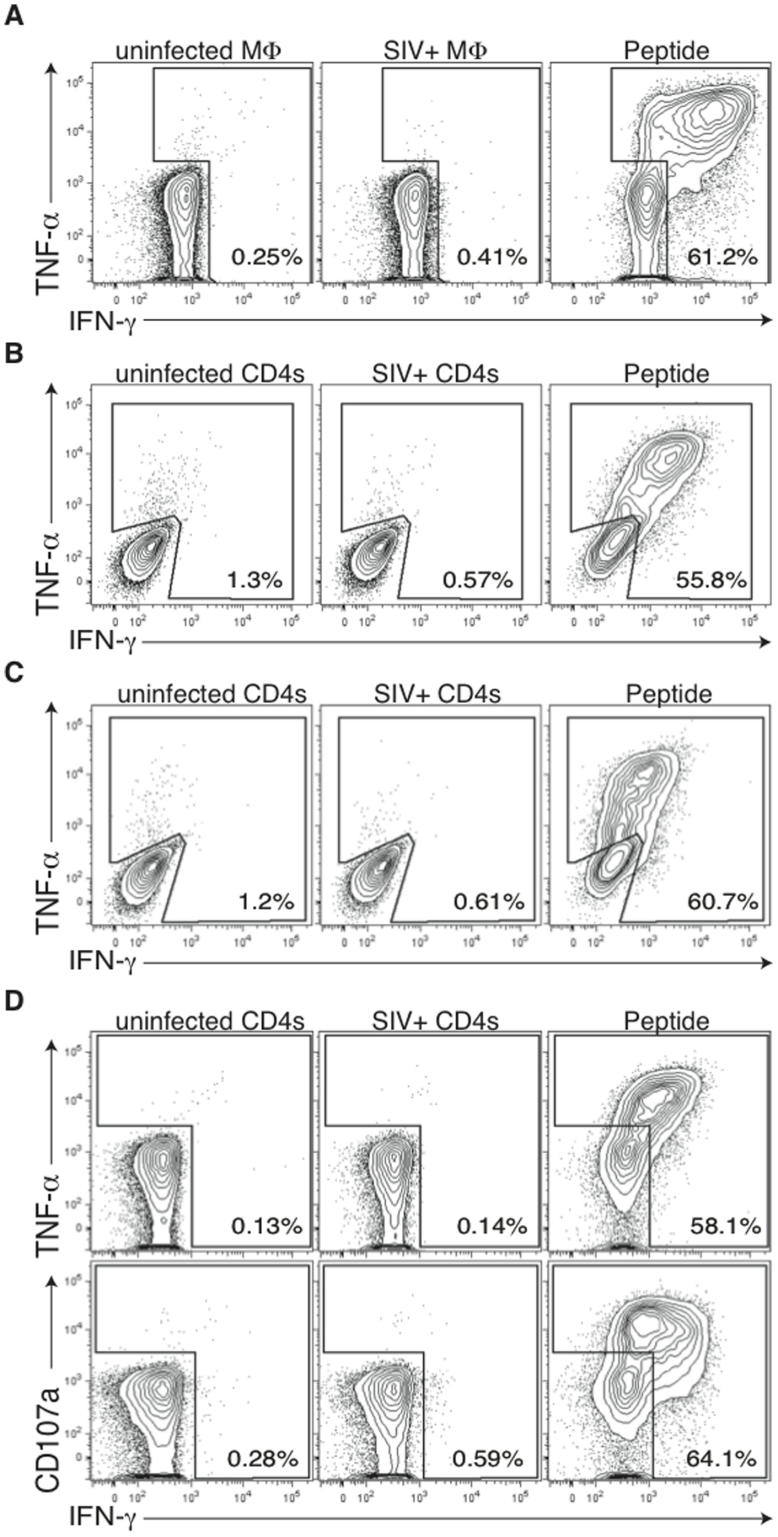
Vaccine induced LINE1 ORF2-, SERV-K Gag-, and SERV-K Env-specific T cells do not recognize SIV-infected cells in vitro. [**A**] An *in vitro-*generated CD4+ T cell line specific for SERV-K Env_667–681/671–685_ NK15/FN15 does not respond to SIV-infected macrophages. [**B**] SERV-K Env_25–33_ LM9-specific CD8+ T cells do not respond to SIV-infected CD4+ T cells. [**C**] LINE1 ORF2_221–229_ RL9-specific CD8+ T cells do not respond to SIV-infected CD4+ T cells [**D**] SERV-K Gag_376–383_ IL8-specific CD8+ T cells do not respond to SIV-infected CD4+ T cells regardless of the cytokine readout [IFN-γ, TNF-α, or CD107a]. Results are indicative of targets infected with either SIVmac239 or SIVsmE660, except for panel A, which is indicative of both SIVsmE660 and SIVmac316E [a macrophage tropic variant of SIVmac239]. Dot plots were generated by gating on CD3+ CD4+ T cells [panel A] or CD3+ CD8+ T cells [panels B-D]. Percentages are indicative of cytokine producing cells. Exogenous peptide antigen was included as a positive control in all recognition assays.

The paucity of anamnestic SERV-K Gag and Env- and LINE-1-specific T cell responses following SIVsmE660 infection indicated a deficiency in the ability of ERE-specific T cells to recognize SIV-infected targets. We therefore sought to determine whether vaccine generated SERV-K- and LINE-1-specific T cells could recognize SIV-infected cells *in vitro*. To this end we generated a panel of SERV-K Gag-, SERV-K Env-, and LINE1-specific T cell lines and then tested these cell lines for recognition of SIV-infected targets. In agreement with the lack of *in vivo* expansion following SIVsmE660 infection, SERV-K Env-specific CD4+ T cells failed to recognize SIV-infected macrophage targets (Fig. 4A) and SERV-K Env-specific CD8+ T cells failed to recognize SIV-infected CD4+ T cell targets (Fig. 4B) as measured by cytokine secretion. Similar results were obtained with LINE-1-specific CD8+ T cells (Fig. 4C), suggesting that SIVsmE660 did not activate LINE-1 expression in macaque cells. Finally, SERV-K Gag-specific CD8+ T cells also did not respond to SIV-infected targets (Fig. 4D). No recognition of SIV-infected cells was observed in with any ERE-specific T cell line tested by either cytokine secretion or degranulation (CD107a) (Fig. 4 and data not shown), further suggesting that vaccine-elicited SERV-K Gag-, SERV-K Env-, or LINE-1-specific T cells do not specifically respond to SIV infection in rhesus macaque cells.

**Figure 5:**
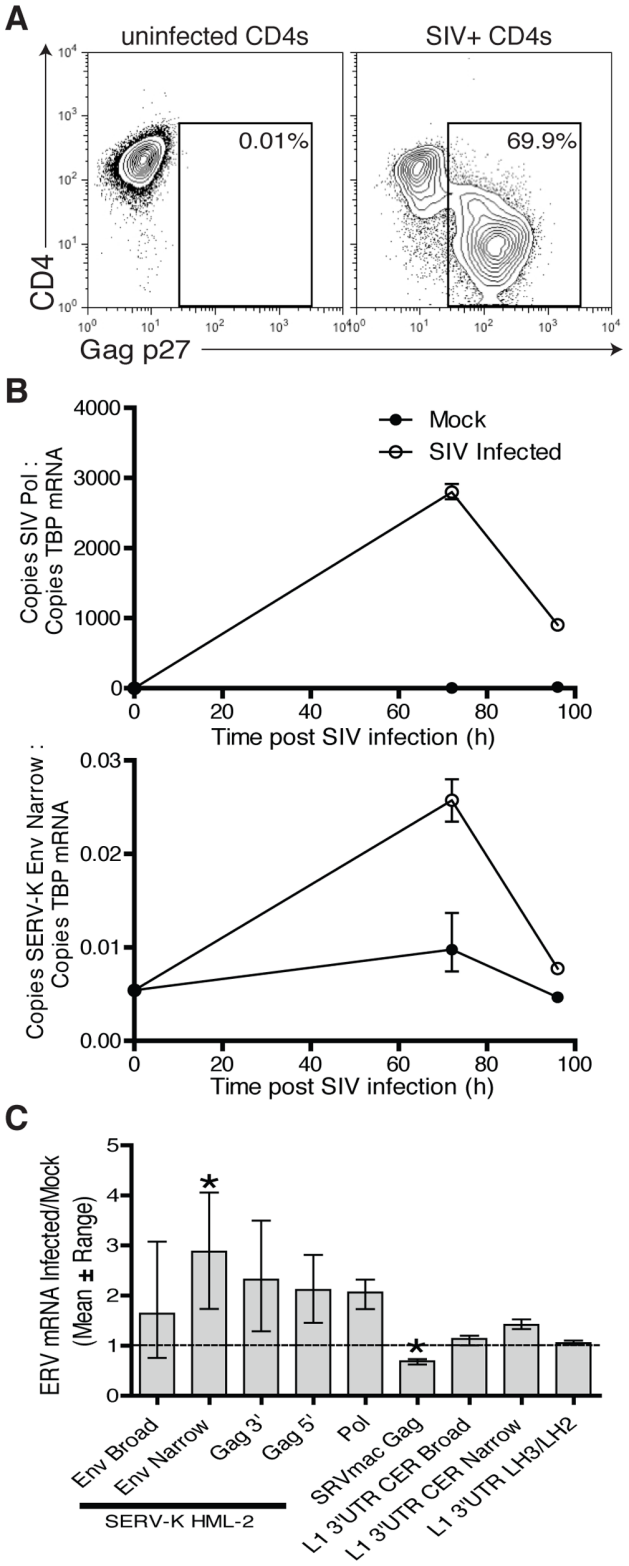
Analysis of ERE mRNA expression in SIV-infected versus uninfected CD4+ T cells. [**A**] Flow cytometry analysis staining for SIV Gag p27 and CD4 of uninfected or SIVsmE660-infected CD4+ T cells used for subsequent qPCR analysis [72 h time point is shown]. [**B**] qPCR analysis of mRNAs of interest in proportion to the housekeeping gene TBP, confirmed SIV infection [top panel] which declined sharply after 72 hours concomitant with a decline in viability of the cell culture. The mRNA levels of the ERE genes of interest were not significantly increased with the exception of SERV-K Env as detected by narrow specificity primers [bottom panel] which showed significant but low level elevation at 72 h. The mean and range at each time point are shown. [**C**] Summary of the qPCR panel by mean and range fold change compared to uninfected cells. Following correction for multicomparisons only the 2.9-fold increase in mRNA of SERV-K Env as detected by narrow specificity primers and the 1.4-fold decrease in SRVmac Gag remained significant.

Based on the inability of our vaccine-induced, ERE-specific T cells to recognize SIV-infected cells we next investigated whether SIV induced expression of these antigens at the mRNA level in rhesus macaque cells, as we and others have previously observed with HERV-K transcripts in HIV-1-infected human cells [10]. To this end, we infected activated CD4+ T cells with SIVsmE660 via magnetofection and used the highly SIV-infected populations at 72 and 96 for qPCR analysis (Fig. 5A). SIV infection peaked by 72 h following infection and declined rapidly thereafter, concomitant with a decline in viability of the cell culture (Fig. 5B, and data not shown). In designing primer pairs for this analysis we sought to both directly quantify the SERV and LINE-1 sequences corresponding to our vaccine antigens, and to make a limited effort to determine whether other similar SERV/LINE-1 sequences, which may not have precisely matched our vaccine antigens, may have been induced at the mRNA level. Thus the ‘SERV-K-Env narrow’ primers target a relatively narrow subset of SERV-K sequences, but align perfectly with the consensus sequence on which the vaccine inserts were based, while the ‘SERV-K-Env broad’ target more broadly – in part due to the incorporation of degenerate nucleotide bases. Similarly, ‘SERV-K-Gag-broad’ targets a broad range of sequences, than ‘SERV-K-Gag-narrow’. The LINE-1 (L1) 3′UTR LH3/LH2 primer pair was selected to match the ‘hot’ LINE-1 insertions, which exhibit evidence of recent activity in the human genome, corresponding with the LINE-1 ORF2 sequence incorporated into our vaccine. The L1-3′UTR CER were selected to amplify the CER-3 and CER-4 subfamilies of LINE-1 which appear to be replication competent in the rhesus macaque genome [16]. We detected small increases in the levels of mRNA between the uninfected and SIV–infected cells at 72 h for a number of ERE primer sets, however after correction for multiple comparisons only the increase in SERV-K Env mRNA, as detected by the narrow-specificity primer set, and the decrease in SRVmac Gag mRNA were statistically significant (Fig. 5C). Compared with levels of HERV-K mRNA induction observed with HIV infection of human CD4 T cells [8] the absolute and fold-levels of induction were substantially lower, suggesting that SIVsmE660 does not meaningfully induce SERV-K or LINE-1 at the mRNA level in rhesus macaque cells.

### TRIM5α Impacts SIVsmE660 Acquisition and Replication

**Figure 6:**
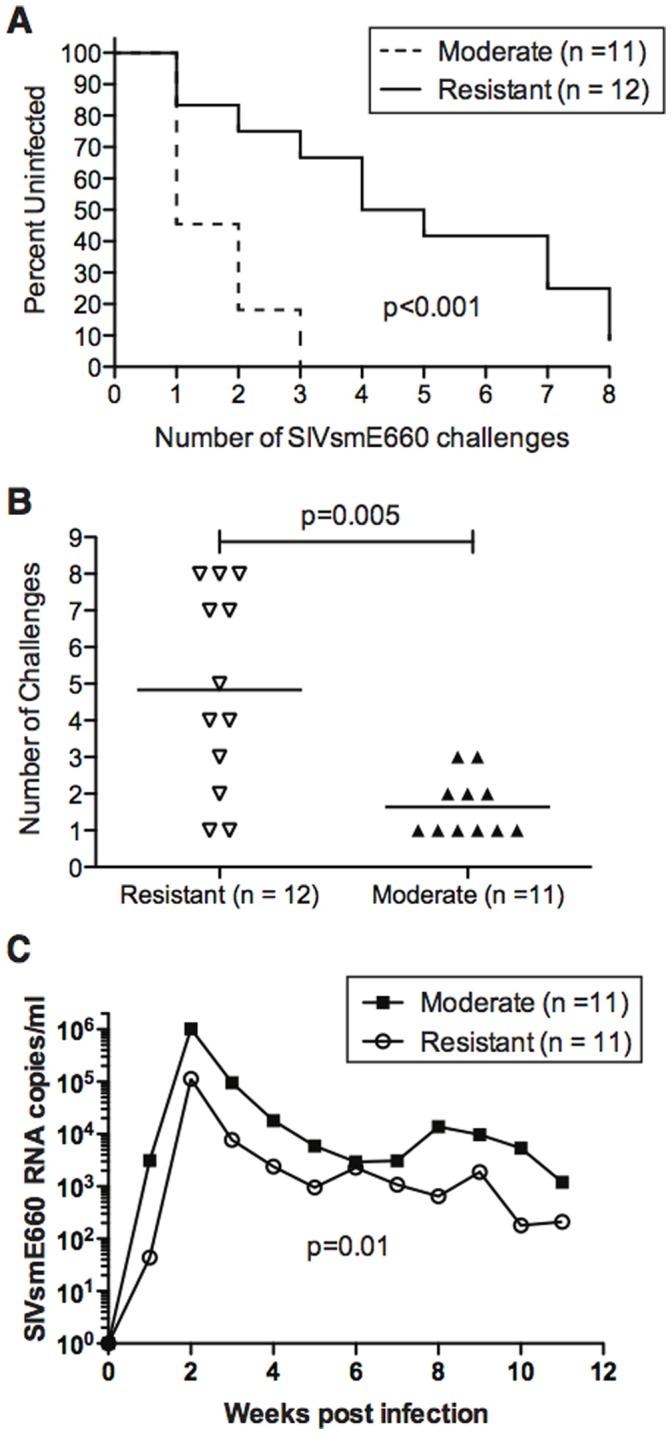
Effect of TRIMα on SIVsmE660 acquisition and replication. Kaplan-Meyer curve analysis of the effect TRIM5α had on the rate of acquisition of SIVsmE660 infection after repeated limiting-dose intrarectal challenge. The statistical significance of the rate of infection was determined by log rank test. Note that animal r99080 was the only animal with a susceptible phenotype based on TRIM5α and therefore was excluded from all TRIM5α analysis. [**B**] Comparison of the number of challenges needed to productively infect animals with SIVsmE660 based on the presence of resistant TRIM5α alleles. The statistical significance of the number of challenges required between the groups was performed by generalized gamma model. [**C**] The geometric mean of the viral loads of each TRIM5α group is shown. A statistically different value was observed between the groups as measured by the area under of the curve. The statistical difference between the groups in area under of curve was performed by one-way ANOVA. Note that animal r07045 was never infected and is, therefore, excluded from this analysis.

Despite finding no effect of our ERE vaccine on SIVsmE660 acquisition and replication, particular animals did resist SIVsmE660 acquisition and maintained low viral loads during infection (Fig. 1B and Fig. 2A–C). Differential *TRIM5* allele expression has been shown to affect the acquisition of SIVsmE543-3 and SIVsmE660 and replication kinetics of SIVsmE543-3 in rhesus macaques [28], [37]–[39]. There are 12 characterized *TRIM5* alleles found in rhesus macaques that can be assigned into one of three functional groups based on their viral capsid binding domains and effect on SIVsmE543-3 replication: *TRIM5^TFP^*, *TRIM5^CypA^*, and *TRIM5^Q^* [28]. TRIM5^TFP^ and TRIM5^CypA^ restrict SIVsmE543-3 replication, both *in vitro* and *in vivo*, while TRIM5^Q^ exhibits no effect [37]. Rhesus macaques co-dominantly express two *TRIM5* alleles and can therefore be grouped as most resistant (*TRIM5^TFP^*/*TRIM5^TFP^*, *TRIM5^TFP^*/*TRIM5^CypA^*, and *TRIM5^CypA^*/*TRIM5^CypA^*), moderately resistant (*TRIM5^TFP^*/*TRIM5^Q^* and *TRIM5^CypA^*/*TRIM5^Q^*), or susceptible (*TRIM5^Q^*/*TRIM5^Q^*) to SIVsmE660 infection. To determine whether *TRIM5* affected intra-rectal SIVsmE660 acquisition or replication within our study, we genotyped for *TRIM5* allele expression and subsequently placed animals in groups based on their *TRIM5* alleles (Table S1). Importantly, the stock of SIVsmE660 used in our study has been deep-sequenced previously and all sequences obtained were identical to SIVsmE543-3 at the Gag positions conferring susceptibility to *TRIM5^TFP^* and *TRIM5^CypA^* [28]. In line with previous reports [28], we found that animals with resistant *TRIM5* genotypes took significantly longer (Fig. 6A) and required more low dose IR challenges to become infected with SIVsmE660 (Fig. 6B). However, it remained possible that our vaccine had a detrimental impact and exacerbated SIV acquisition as previously described [40]. To examine this possibility, the control and vaccine groups were reexamined for viral acquisition according to resistant *TRIM5* genotype. No statistically significant difference was observed between ERE vaccinated and unvaccinated animals expressing the same protective *TRIM5*α alleles (Figure S1), suggesting that the vaccine had no measurable effect on the speed with which animals became infected. Additionally, we observed significantly lower viral loads in the resistant group compared to the moderately resistant group following SIVsmE660 infection (Fig. 6C). It is important to note that the only animal with a susceptible *TRIM5* genotype (r99080) became infected after only a single challenge with SIVsmE660 and maintained high viral loads through the first twelve weeks of infection (Fig. 2A). Therefore, it appears that the *TRIM5* genotype of animals in vaccine studies can have a significant impact on both acquisition and replication of SIVsmE660.

## Discussion

Antigens derived from a number of specific EREs are expressed in a range of tumors [41]–[54]. Although contradictory reports exist on the relationship between HERV-K activation and HIV replication [55], [56], we, and others, have also found selected antigens to be associated with HIV infection [2]–[8]. Thus vaccination with ERE antigens represents a novel strategy for targeting host immunity to tumors or HIV-infected cells. While testing the safety and immunogenicity of SERV-K Env & Gag, and human LINE-1 in rhesus macaques to enable further development of ERE-targeting strategies in both cancer and HIV infection, we reasoned that the rhesus macaque SIV challenge model might also be suitable for assessing their efficacy as stable surrogate targets marking SIV-infected cells. We therefore investigated the levels of mRNA for SERV-K and LINE-1 in primary SIV-infected macaque CD4 T cells. This investigation revealed only a slight increase in the levels of mRNA for the targeted SERV-K sequences and no induction of targeted LINE-1 sequences. The levels of SERV-K induction were substantially lower than we had seen in human cells infected with HIV [8]. Indeed, with the largest change being <3 fold increase, regulation at the mRNA level is not likely to trigger an SIV-dependent ‘on/off’ expression of these ERE proteins as we have seen in humans. These results are in agreement with a recent study demonstrating that SIV does not induce the expression of cynomolgous macaque ERV expression at the mRNA level [57]. However, repressive mechanisms targeting EREs act at many levels both pre- and post-transcription [14] [15] [22] [24], and it is not clear how HIV/SIV triggers ERE expression, therefore changes in mRNA levels might not relate to changes in SERV-K and L1 translation. To explore this further, we isolated SERV-K and L1O2-specific T cell clones from macaques vaccinated in our safety study [13], but these failed to respond to SIV-infected cells, whereas they made strong responses to cognate peptide. Finally, we challenged the macaques with SIV via the repeated low dose intrarectal route until they became infected, with the aim of culling the animals for further safety investigation 10–12 weeks following first positive SIV blood sample (before the onset of clinically-apparent pathology). We observed that the control and vaccinated groups had indistinguishable susceptibility to infection and viral loads post infection, thus no efficacy was seen with the ERE-vaccination approach. Differences in infection rate and viral loads occurred, but were mediated by differences in TRIM-5α genotype. We were able to follow the T cell response to 20 ERE-derived epitopes across six animals during the challenge phase in order to expand upon our *in vitro* observations as to whether SIVsmE660 might induce SERV-K or LINE-1 expression. None of the immune responses that we tracked were boosted by SIV infection, suggesting a lack of recognition in all cases. Together with the mRNA and T cell clone data we conclude that the Indian rhesus macaque SIVsmE660 model may not be suitable to test the efficacy of vaccination-induced immune responses to SERV-K Env & Gag, and human LINE-1 ORF2 at preventing or controlling SIV infection. However, one major caveat of the current study is the use of consensus ERE sequences corresponding to the HML-2 family in humans as vaccine sequences [15] [13]. Each species contains a unique set of EREs that may not be regulated analogously to other closely related species. For example, HERV-K111 is a novel HML-2 provirus that is specifically activated by HIV infection in human cells, likely through the activity of the viral Tat protein [58]. Thus the HERV-K111 provirus represents an ideal target for testing the ERE vaccine concept to protect from AIDS viruses. However, HERV-K111 proviruses are absent from other primate species, with the exception of chimpanzees [58], precluding direct translation of this human-specific ERE to pre-clinical animal safety and efficacy studies. Furthermore, the use of consensus ERE sequences as vaccine targets would not induce cellular responses against small, degraded EREs. Robust, effective T cell responses in the setting of both HIV infection [5] and cancer [59], [60] have been identified against truncated, highly mutated ERE ORFs that would not be expected to code for epitopes. Therefore, the current vaccine results could also be the result of targeting the wrong EREs in the macaque model. Indeed, the major remaining hurdle to testing the efficacy of ERE-specific immune responses against HIV infection is that not only do the infecting viruses vary between humans (HIV) and nonhuman primates (SIV), but also the genomic constellation of EREs present in each species. We propose that the path forward for ERE antigens as potential HIV vaccine candidates consists of several options, including: the search for other ERE antigens that are induced by SIV in rhesus, cynomolgus, or pigtail macaques [61]; the potential use of HIV-2 in pigtail macaques [61], or novel HIV-1-derivatives in pigtail or cynomolgus macaques [62]–[64] [65], [66]; the search for feline EREs induced by FIV in the feline challenge model [66]; or finally, the use of a suitable humanized mouse model such as the BLT mouse [67], [68]. The BLT mouse model is particularly attractive as it would enable the testing of the ERE approach using human ERE targets and HIV, but safety aspects relating to off-target recognition of many non-immune tissues could not be studied in this model. Instead information on safety aspects of potential autoimmunity other than towards the human bone marrow, liver, and thymus, would need to come from analogous studies in macaques, such as the initial safety study we have already completed [13].

There is growing evidence and appreciation of the interaction between germline ERE and exogenous retroviruses in multiple species. For example, infection of mice with exogenous ectopic MuLV results in replication of defective endogenous polytopic retroviruses [1]. In HIV-infected humans, multiple groups have reported activation of ERE during viral replication [3]–[11] [69]. Thus, targeting host immunity to HIV-1-infected cells based on expression of specific ERE is a novel approach to circumvent the problem of viral diversity. Although we did not prevent viral acquisition or replication in the current study, we still believe the approach of targeting ERE to prevent HIV-1 replication is viable and should be explored further. To this end, we propose that further studies on the interaction between ERE and exogenous retroviruses in multiple animal models of HIV infection are warranted.

## Supporting Information

Figure S1 Vaccine status does not accelerate SIV acquisition. Kaplan-Meyer curve analysis of the effect TRIM5 had on the rate of acquisition of SIVsmE660 infection after repeated limiting-dose intrarectal challenge with animals stratified by **(A)** moderately resistant TRIM5α alleles, or **(B)** the most resistant TRIM5α alleles. The statistical significance of the rate of infection was determined by log rank test. No statistically significant difference is observed (NS = Not Significant). Comparison of the number of challenges needed to productively infect animals with SIVsmE660 based on the presence of **(C)** moderately resistant TRIM5 alleles, or **(D)** the most resistant TRIM5 alleles. The statistical significance of the number of challenges required between the groups was performed by generalized gamma model. No statistically significant difference is observed (NS = Not Significant).
(TIFF)

Table S1 Animals used in the ERE vaccine study. Sex, MHC-I genotype, and TRIM5 genotype, and grouping are shown for all 24 animals in the study. *r07045 remained uninfected following five low dose IR challenges, one medium dose IR challenge, one high dose IR challenge, and one IV challenge with SIVsmE660.
(TIFF)

Table S2 Standards used for ERE mRNA studies. The indicated sequences were synthesized and cloned into pUC57. Standard curves were generated from serial dilutions of linearized plasmids to establish absolute quantifications.
(TIFF)

Table S3 Primers used for ERE mRNA studies.
(TIFF)
