# Supplementary figures and images for: Vaccination against Endogenous Retrotransposable Element Consensus Sequences Does Not Protect Rhesus Macaques from SIVsmE660 Infection and Replication
Source: PLoS One. 2014 Mar 20;9(3):e92012. doi: 10.1371/journal.pone.0092012 (PMC3961289; doi:10.1371/journal.pone.0092012)

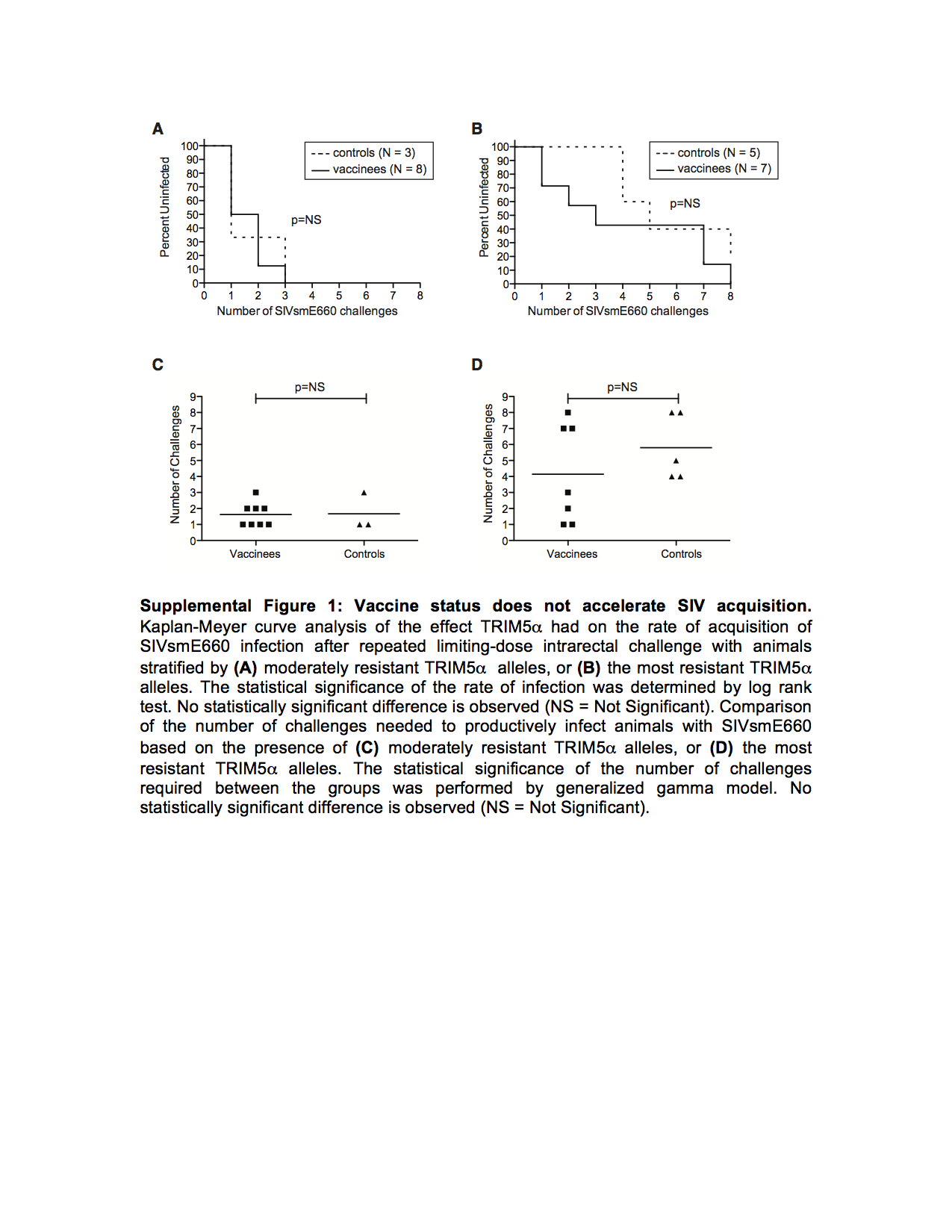

Supplement: Figure S1 — Vaccine status does not accelerate SIV acquisition. Kaplan-Meyer curve analysis of the effect TRIM5 had on the rate of acquisition of SIVsmE660 infection after repeated limiting-dose intrarectal challenge with animals stratified by (A) moderately resistant TRIM5α alleles, or (B) the most resistant TRIM5α alleles. The statistical significance of the rate of infection was determined by log rank test. No statistically significant difference is observed (NS = Not Significant). Comparison of the number of challenges needed to productively infect animals with SIVsmE660 based on the presence of (C) moderately resistant TRIM5 alleles, or (D) the most resistant TRIM5 alleles. The statistical significance of the number of challenges required between the groups was performed by generalized gamma model. No statistically significant difference is observed (NS = Not Significant). (TIFF) [file pone.0092012.s001.tiff]

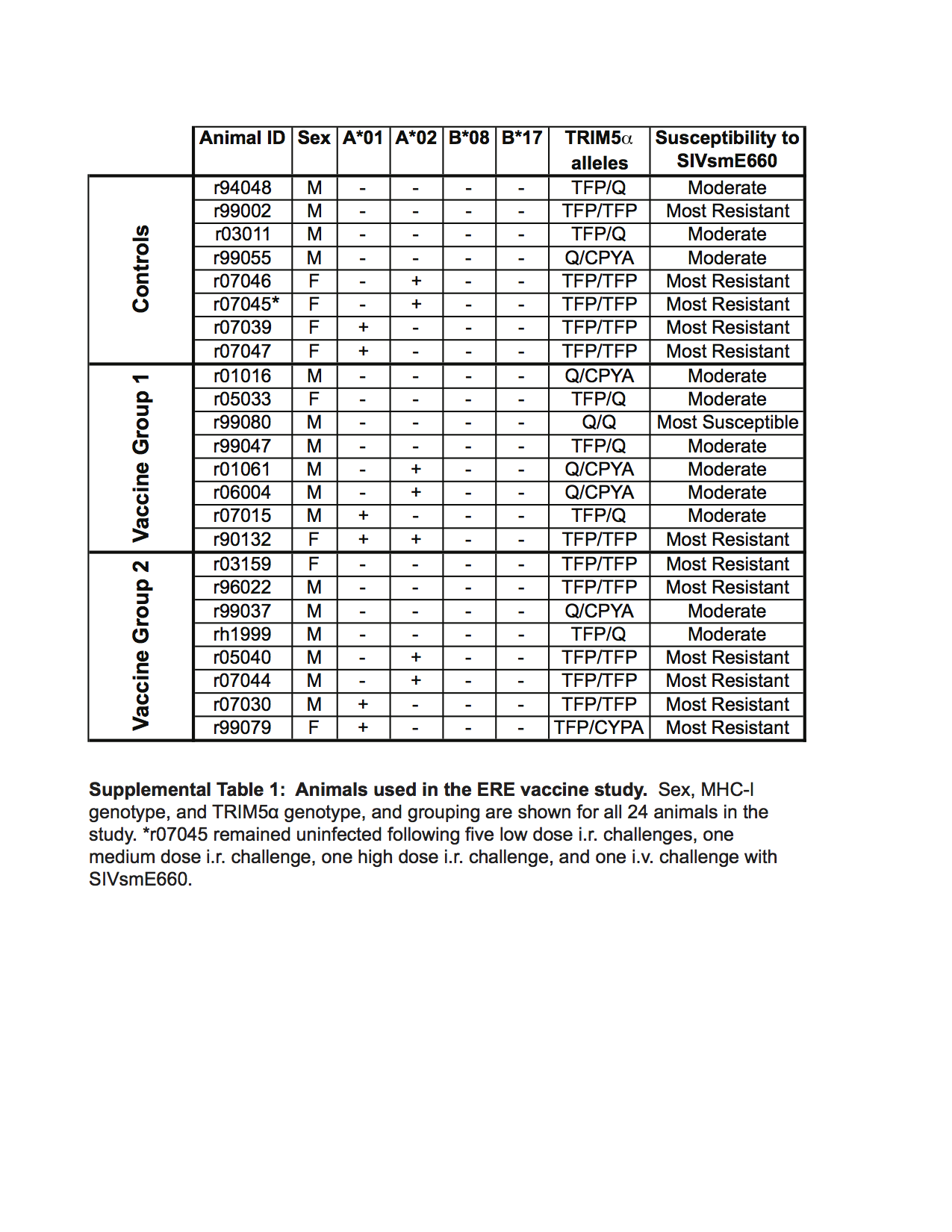

Supplement: Table S1 — Animals used in the ERE vaccine study. Sex, MHC-I genotype, and TRIM5 genotype, and grouping are shown for all 24 animals in the study. *r07045 remained uninfected following five low dose IR challenges, one medium dose IR challenge, one high dose IR challenge, and one IV challenge with SIVsmE660. (TIFF) [file pone.0092012.s002.tiff]

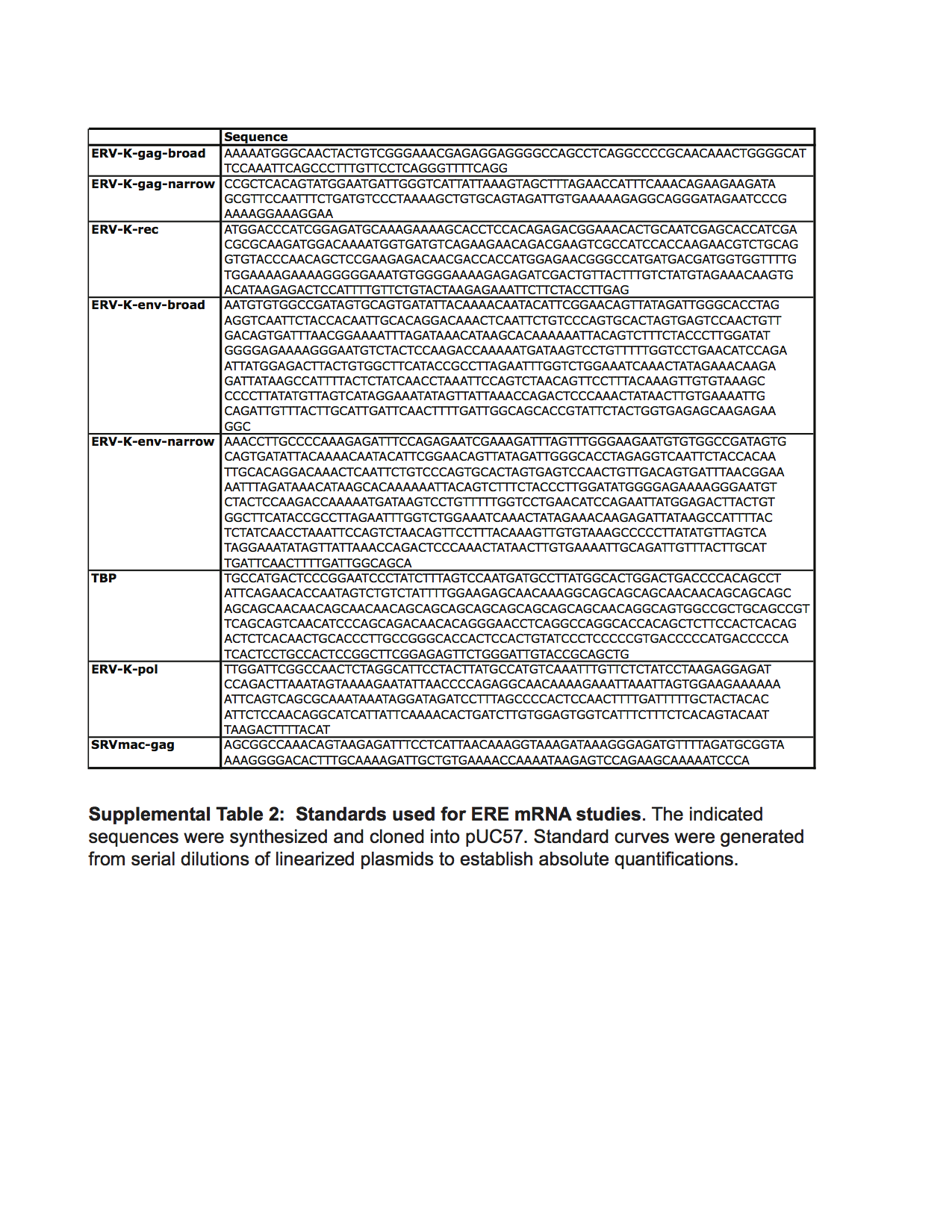

Supplement: Table S2 — Standards used for ERE mRNA studies. The indicated sequences were synthesized and cloned into pUC57. Standard curves were generated from serial dilutions of linearized plasmids to establish absolute quantifications. (TIFF) [file pone.0092012.s003.tiff]

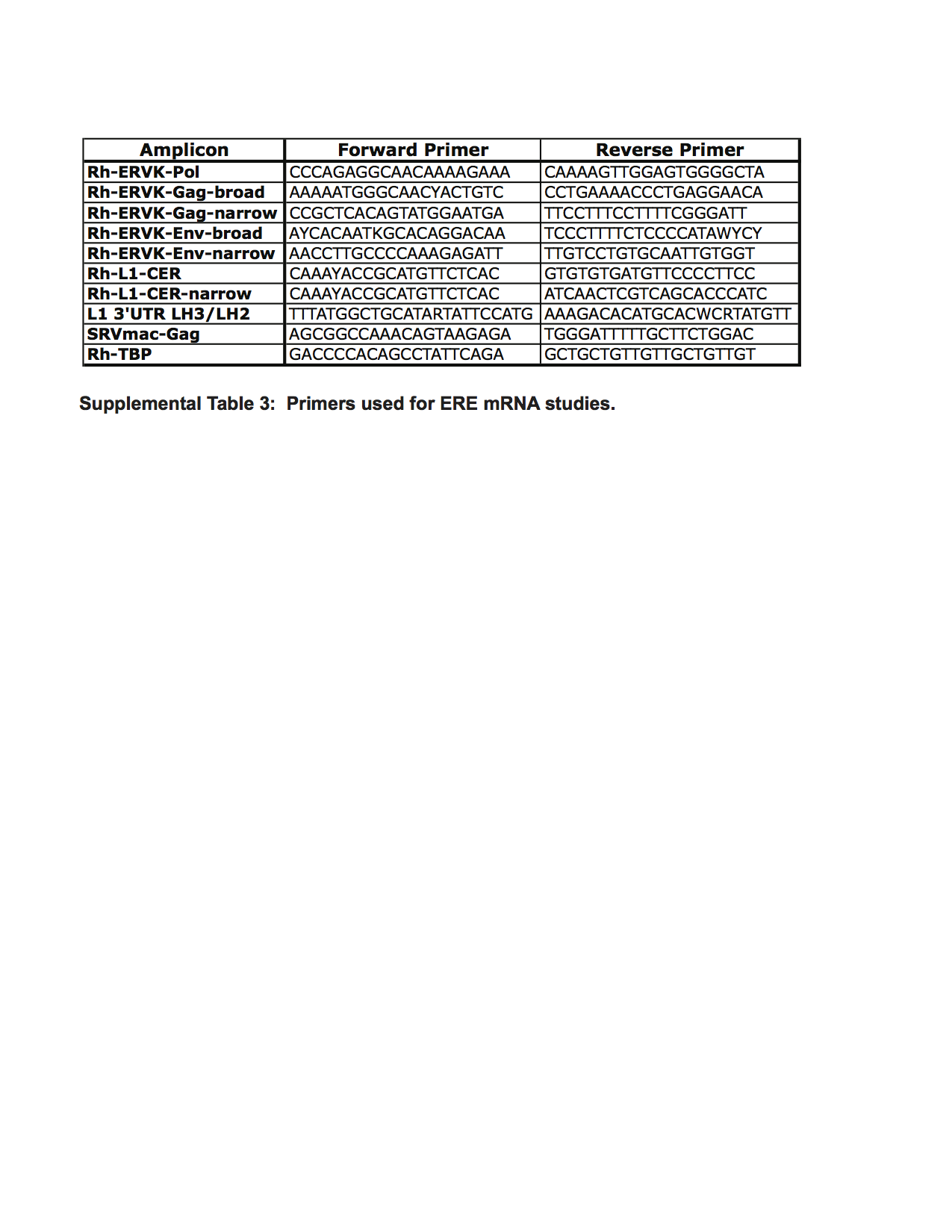

Supplement: Table S3 — Primers used for ERE mRNA studies. (TIFF) [file pone.0092012.s004.tiff]
